# Balance and motion coordination parameters can be improved in patients with type 2 diabetes with physical balance training: non-randomized controlled trial

**DOI:** 10.1186/s12902-021-00804-8

**Published:** 2021-07-03

**Authors:** Artur Stolarczyk, Igor Jarzemski, Bartosz M. Maciąg, Kuba Radzimowski, Maciej Świercz, Magda Stolarczyk

**Affiliations:** 1 Department of Orthopedics and Rehabilitation, Medical University of Warsaw, Warsaw, Poland; 2 Międzyleski Specialist Hospital, Str. Bursztynowa 2, 04-749 Warsaw, Poland; 3 3rd Clinic of Internal Medicine and Cardiology, Medical University of Warsaw, Warsaw, Poland

**Keywords:** Diabetes mellitus, Balance, Fall risk, Physical training, Coordination

## Abstract

**Background:**

Type 2 diabetes (T2D) is a cause of multiple complications, including retinopathy and peripheral neuropathy. These complications are well understood and believed to contribute to gait instability. Poor balance control and increased falling risk have also been reported in people with diabetic peripheral neuropathy (DPN).

Patients with DPN have increased risk of falling due to decreased proprioceptive feedback. Effective balance training should improve postural control in patients with DPN. For this purpose further evaluation was conducted and balance training was designed.

**Methods:**

The goal of our study was to determine values of proprioception, balance, muscle coordination and strength in patients with T2D and analyze whether biofeedback balance training with use of the Biodex Balance System could improve these parameters. To assess the fall risk the general stability index (GSI), the index of frontal-posterior (FPI) and medial–lateral (MLI) stability were evaluated. 37 patients with diagnosed type 2 diabetes mellitus were recruited to this study. Their results were compared with control group consisting of 41 healthy participants who were homogenic to the study group in terms of age and body mass index (BMI).

**Results:**

There were statistically significant differences between patients with diabetes compared to healthy subjects in GSI (2.79 vs 1.1), FPI (1.66 vs 0.7), MLI (0.88 vs 0.52) and risk of falling (5.18 vs 2.72) *p* < 0.05. There were also statistically significant changes before and after training in all stability indices (GSI: 2.79 vs 1.26, FPI: 1.66 vs 0.77, MLI: 0.88 vs 0.54 accordingly) *p* < 0.05 and risk of falling (5.18 vs 3.87) *p* < 0.05 in the study group who had undergone training with biofeedback.

**Conclusions:**

This study found that there is a decreased balance and motor coordination and an increased risk of falling in patients with type 2 diabetes. These parameters improved in patients who have undergone training programme with biofeedback. Furthermore, an age-dependent deprivation of static balance was observed along with an increased risk of falling as a result of increasing BMI.

## Background

Due to the sociological and work-related changes all around the world it is estimated that by the beginning of 2030, the number of cases of Type 2 Diabetes (T2D) will grow from 6059 up to 7079 per 100,000 people [1]. There are studies proving that cardiovascular complications related to T2D are responsible for 4 million deaths annually [2]. The newest epidemiological data suggest that growing number of T2D cases is no longer a problem in developed countries only but it also affects the developing ones. In research performed in Uganda investigators reported that 22.8% of population between 45 and 80 years of age might be affected with this condition. As potential risk factors authors reported alcoholism, nicotinism, high body-mass index (BMI) and family history [3].

Diabetes mellitus may cause several different complications during the course of the disease. One of the most common is peripheral neuropathy (DPN) causing damage to the peripheral sensory and motor nerves even in mild to moderate course of the disease [4]. It is well proved in the literature, that diabetes mellitus has a negative impact on function of the peripheral nervous system by damaging its sensory fibers with age and male sex being the most important risk factors [5].

High risk of falls was reported in the population with diabetes, with an overall incidence of 1.25 falls/person-year [6]. It was proved that short-period strength and balance exercises do not improve the quality of life of patients with diabetes. However, these excercises have a positive impact on the functional outcome in this group [7, 8].

Influence of diabetes mellitus type II (T2D) on motor system is not limited only to the peripheral nervous system. It also affects structures of cerebrum, what leads to changes in projection tracts. In cerebellum it affects the vermis and parts of lobes responsible for receiving impulses from the spinal cord and controlling proximal parts of muscles, which are crucial for movement coordination during gait. Disorders caused by T2D in basal nuclei result in lengthening muscle response time and slower gait velocity [9].

Pharmacological and dietary interventions, together with physical activity are considered the cornerstones of proper T2D management [10]. In addition to the beneficial effect of exercise interventions on glycemic control and lowering the cardiovascular risk factors associated with T2D, physical exercise is effective in improving muscle strength, power output, cardiovascular function and functional capacity in elderly patients with diabetes [11, 12]. In elderly with severe functional aggravation multicomponent exercise programs composed of resistance, endurance, balance, and gait training should be introduced in order to increase the functional capacity and quality of life and to avoid disability and falls [13].

The aim of this study was to evaluate balance and motor coordination parameters in patients treated for T2D who underwent biofeedback-equivalent training with use of the Biodex dynamometric platform.

The hypothesis was that there will be an improvement in the above-mentioned parameters in patients with type II diabetes who were submitted to the feedback-based training using the Biodex platform.

## Methods

### Study design

All subjects were patients of the Department of Internal Medicine and Diabetology of the Medical University of Warsaw, who underwent standard diabetes control visit in January 2020. The control group consisted of patients at least 6 weeks after non-operative distal radius fractures treatment.

Inclusion criteria for the study group were willingness to participate in the study, age over 65, being diagnosed with T2D and being subjected to pharmacological treatment with use of insulin or metformin. For the control group inclusion criteria was not having T2D. Exclusion criteria comprised surgical intervention in the lower limbs or spine during the last 6 months, symptoms of osteoarthritis or pain of other origin in the lower limbs or spine region, rheumatic diseases (e.g., rheumatoid arthritis, ankylosing spondylitis), diagnosed neuromuscular disease, strongly manifested imbalances due to impairment of central or peripheral nervous system and neurological disorders with dizziness, nystagmus or serious neurological symptoms (cerebrospinal syndrome, multiple sclerosis, Parkinson's disease, etc.), taking medications that could impact stability, cardiovascular disorders (including stroke or transient ischemic attack in the past).

All participants in the study group were subject to a 3-month intention-to-treat balance training. Measurements were made right before and immediately after training. In the control group, the tests were performed once to determine baseline parameters for proprioception and balance for a given age group. To avoid risk of bias information of potential T2D was blinded for the assessor.

This study is reported in accordance with the Preferred Reporting Items for Consolidated Standards of Reporting Trials (CONSORT) statement.

#### Intervention

The patients were subject to a proprioception, balance and motor coordination training with use of the dynamic platform—Biodex Balance System (Biodex 945-302, Biodex Medical Systems Inc., Shirley, New York). The platform software includes ready-to-use exercise protocols that are simple games involving moving the center of gravity of the body in a specific way in both static and dynamic settings. These games are based on a biofeedback mechanism and require conscious adjusting of the body position according to a spot on the monitor that corresponds to the subjects own center of gravity. Biodex Balance System is considered as a reliable and objective tool for balance assessment and training [14].

Training sessions were held daily for 3 months, excluding the weekends. The duration of a single training session was 30 min. The difficulty of training was chosen individually for the needs of the patient. Initially, the training was done on a stable basis. As the patient trained, the difficulty of the exercise increased, adding exercises on an unstable substrate. In addition, the task required a more precise control of the center of gravity.

Evaluation of balance and motion coordination parameters was performed using the Biodex Balance System platform, which makes it possible to evaluate the aforementioned parameters in statics and dynamics. Each patient performed three tests: a biofeedback posture test with open and closed eyes and a fall risk test. The posture stability test consisted in maintaining the center of gravity at one point with a stable substrate. Three stability measures were identified based on three 20-s balance attempts: General Stability Index (GSI), FPI (Frontal-Posterior Stability Index) and MLI (Medial–Lateral Stability Index). The smaller the value of the indices, the better the test score. The test was performed with opened and closed eyes.

In the fall risk (RF) test using the Biodex platform, the subject was told to maintain the center of gravity on an unstable substrate in three 20-s trials. The lower the values of the fall index were, the better the result.

#### Methods of statistical analysis

Statistica 13.1 programme (StatSoft, Tulsa, Okla, United States) was used for statistical analysis of the obtained results. As the variables did not have a normal distribution, the non-parametric Mann–Whitney test was used to compare the results of the control group with the study group. In order to compare the results within the group before and after the training, the non-parametric Wilcoxon test for paired variables was used. The threshold of statistical significance was assumed to be 0.05. In addition, the upper and lower limits of the 95% confidence interval for the mean difference were determined. A correlation analysis was also performed to examine the linear relationship between the variables. For each correlation the strength of the relationship was determined: weak, moderate or strong. Correlation coefficient values from 0 to 0.3 were described as a weak correlation, values from 0.3 to 0.5 as moderate and values from 0.5 to 1 as strong. As the variables had no normal distribution, the correlations were calculated on the basis of the nonparametric Spearman coefficient.

## Results

There were 77 participants in the study. The study group consisted of 37 patients diagnosed and treated with T2D. 41 patients were recruited to the control group. No one resigned or was removed from study.

There were 18 men and 19 women in the study group with mean age of 73.22 (SD = 7.57). The average BMI was 29.5 kg/m^2^ (SD = 4.5), with 5 people having normal body mass (BMI = 18.9–24.9 kg/m^2^), 17 being overweight (BMI = 25–29.9 kg/m^2^), 12 with first degree obesity (BMI = 30—34.9 kg/m^2^), 1 person with second degree obesity (BMI = 35–39.9 kg/m^2^) and 2 people with third degree (BMI > 40 kg/m^2^). Mean time from the diagnosis of T2D was 9.3 years (SD = 2.45). 27 of participants (73%) had hypertension, 25 (67.6%) had hypercholesterolemia, none of them had retinopathy nor neuropathy (Table 1).

**Table 1:** Descriptive statistics for group characteristics

	Study group *n* = 37	Control group *n* = 41	*p*
Sex	18 males, 19 females	17 males, 24 females	0.456
Age (years)	73.22 (60–86)	74.10 (67–80)	0.515
Height (m)	1.68 (1.48–2)	1.69 (1.53–1.82)	0.920
Weight (kg)	84.35 (42–133)	76.71 (53–99)	**0.049**
BMI (kg/m^2^)	29.54 (19.17–41.00)	26.88 (20.76–30.81)	**0.001**
Normal weight	5	7	
Pre-obesity	17	30	
Obesity class I	12	4	
Obesity class II	1	0	
Obesity class III	2	0	

Where applicable results presented as mean (range). BMI classes as per WHO

### Stability test of posture with biofeedback

The results of the assessment of balance and movement coordination parameters using the postural stability test under eye control are presented in Table 2.

**Table 2:** Biofeedback posture test results

	GSI			FPI			MLI		
	C	A1	A2	C	A1	A2	C	A1	A2
Median	1.0	2.7	1.2	0.7	1.5	0.7	0.5	0.8	0.5
Min–max	0.8–2.0	2.1–4.6	0.7–2.0	0.2–1.0	0.9–3.9	0.2–2.0	0.3–0.8	0.4–2.2	0.3–1.0
*p *value									
C versus A1	**< 0.001**			**< 0.001**			**< 0.001**		
A1 versus A2		**< 0.001**			**< 0.001**			**< 0.001**	

C—control group; A1, A2—study group, before and after intervention

Statistical analysis using the Mann–Whitney *U* test showed significantly worse results for all three stability indices in patients with diabetes compared to healthy subjects. For the general stability index and the index of frontal-posterior and medial–lateral stability, the *p* value was less than 0.001.

**Fig. 1:**
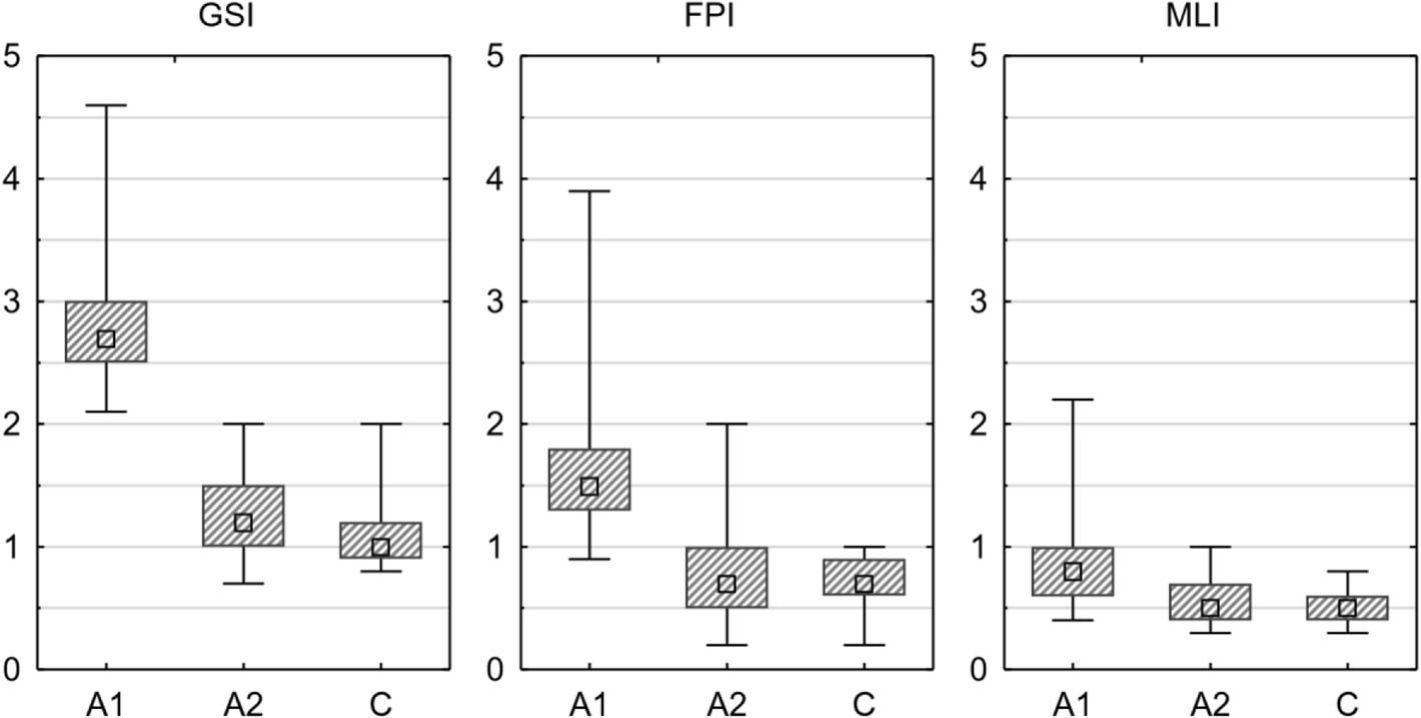
Box and whiskers graphs of results for the biofeedback posture test. C—control group; A1, A2—study group, before and after intervention. Square—median, box—25–75%, whiskers—min–max

Testing Wilcoxon couples showed significant improvement in static balance with visual control in subjects with type 2 diabetes (study group) who received training with biological feedback. A significant improvement was observed for all 3 stability indices with *p* < 0.001 (Fig. 1).

### Posture stability test with closed eyes

In Table 3 the descriptive statistics for the pre- and post-training group and descriptive statistics for the control group were presented.

**Table 3:** Biofeedback posture test with closed eyes results

	GSI			FPI			MLI		
	C	A1	A2	C	A1	A2	C	A1	A2
Median	2.3	4.5	3.1	1.1	3	2	0.5	1.1	0.7
Min–max	1.4–2.9	3.2–5.5	3.0–4.3	0.7–1.6	1.0–4.9	0.7–2.9	0.3–0.9	0.5–2.0	0.4–1.6
*p* value									
C versus A1	**< 0.001**			**< 0.001**			**< 0.001**		
A1 versus A2		**< 0.001**			**< 0.001**			**< 0.001**	

C—control group; A1, A2—study group, before and after intervention

**Fig. 2:**
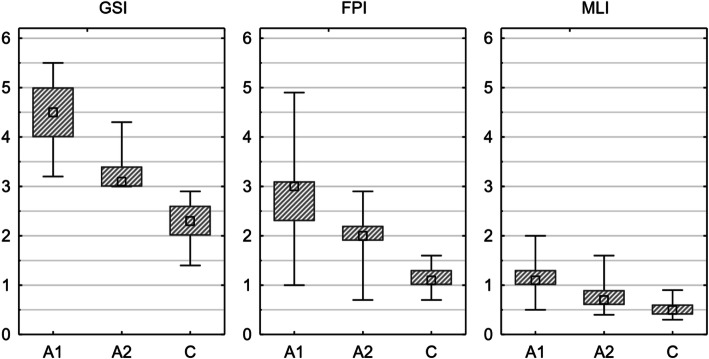
Box and whiskers graphs of results for the biofeedback posture test with closed eyes. C—control group; A1, A2—study group, before and after intervention. Square—median, box—25–75%, whiskers—min–max

Analysis of the results showed statistically significant disturbances in the parameters of balance and motor coordination in group B patients in relation to group K. Furthermore, significant differences in the general, frontal-posterior and medial–lateral stability index (*p* < 0.0001). Analysis of the results obtained from the study group before and after 3 months of training showed significant improvement (*p* < 0.001) for all three stability indicators. The results are shown in Fig. 2.

### Fall risk test

Table 4 represents the statistical analysis for two groups of participants for a fall risk test (RF), which consisted of maintaining the center of gravity at one point on an unstable substrate.

**Table 4:** Fall risk test results (RF)

	C	A1	A2
Median	2.8	5	3.7
Min–max	2.0–3.4	3.8–7.1	3.0–5.4
*p* value			
K versus B1	**< 0.001**		
B1 versus B2		**< 0.001**	

C—control group; A1, A2—study group, before and after intervention

**Fig. 3:**
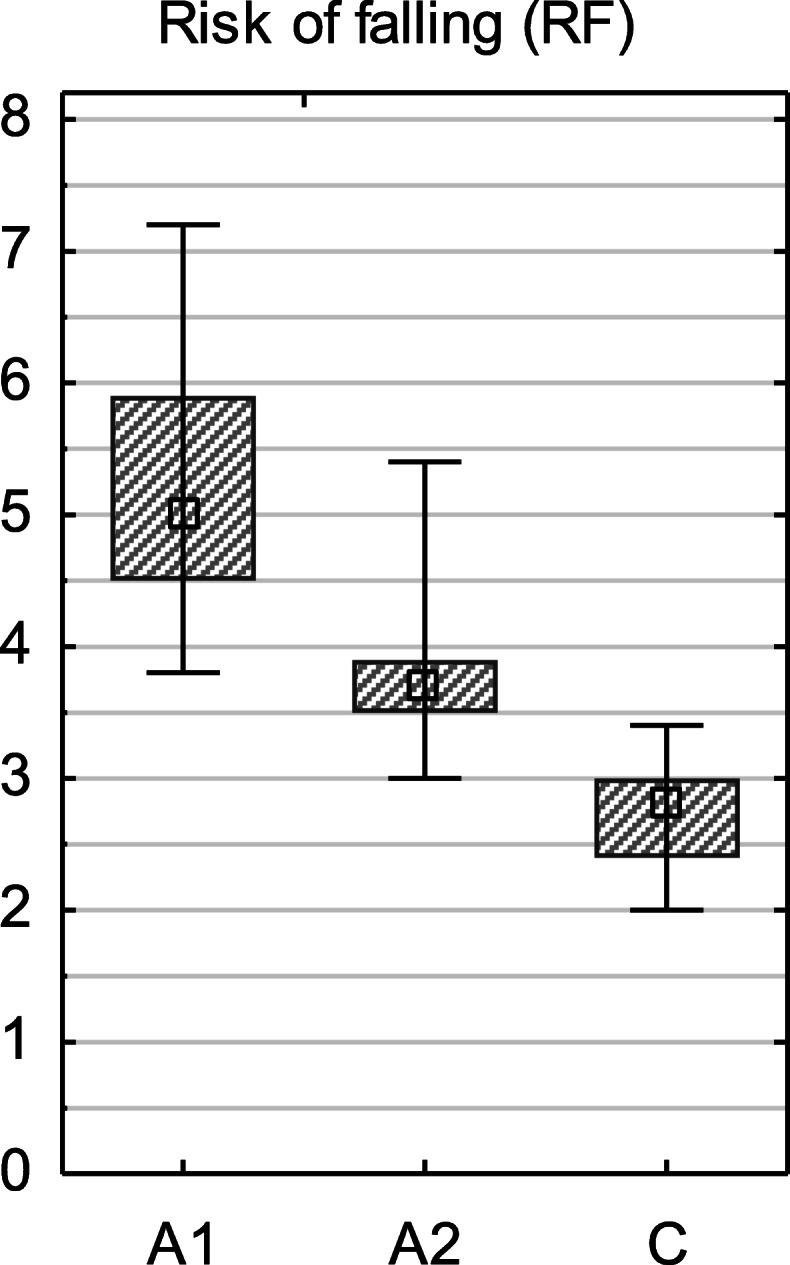
Box and whiskers graphs of results for the risk of falling test. C—control group; A1, A2—study group, before and after intervention. Square—median, box—25–75%, whiskers—min–max

The Mann–Whitney U test showed a statistically significant increase in the risk of falling in people with diabetes compared to healthy individuals (Fig. 3). Statistically significant decrease in the risk of falling was observed in the study group who had undergone training with biofeedback (*p* < 0.001).

### Correlations

In Table 5. Spearman rank correlation results are shown for the study group prior to the training session. Significant correlations were found, where the significance factor p was less than 0.05. There was a correlation between the age of patients and the values of general and anterior–posterior stability index during the postural stability test. With the increasing age, the value of GPI, FPI and MLI index values increased during post-biofeedback posture testing. It was observed that with the increase in BMI, the risk of falling in people with diabetes increased.

**Table 5:** Spearman rank correlations (*p* < 0.05) for the study group prior to the training session

	Age	BMI	GPI BF	FPI BF	MLI BF	GPI CE	FPI CE	MLI CE	RF
Age	1	0.04	0.33	0.62	0.13	0.22	0.22	0.44	0.23
BMI	0.04	1	− 0.08	0	− 0.22	0.01	0.18	0	0.41
GPI BF	0.33	− 0.08	1	0.26	0.57	0.28	− 0.2	0.09	− 0.13
FPI BF	0.62	0	0.26	1	0.07	0.25	0.37	0.27	− 0.14
MLI BF	0.13	− 0.22	0.57	0.07	1	− 0.08	− 0.27	− 0.18	− 0.09
GPI CE	0.22	0.01	0.28	0.25	− 0.08	1	0.4	0.01	− 0.05
FPI CE	0.22	0.18	− 0.2	0.37	− 0.27	0.4	1	− 0.05	− 0.12
MLI CE	0.44	0	0.09	0.27	− 0.18	0.01	− 0.05	1	0.3
RF	0.23	0.41	− 0.13	− 0.14	− 0.09	− 0.05	− 0.12	0.3	1

BF—biofeedback posture test, CE—posture test with closed eyes

The assessment of the relationship between individual characteristics of patients with diabetes who were subject to balance training with the help of the Biodex platform is presented in Table 6. It was observed that with increasing age, balance parameters in a static setting with opened and closed eyes and the mean muscle strength, for both knee flexors and extensors were decreased. Analysis showed that with the deterioration of static balance during biofeedback testing, the strength of the tested muscle groups decreased. Patients with greater BMI showed an increased risk of falling.

**Table 6:** Spearman rank correlations (*p* < 0.05) for the study group after the training session

	Age	BMI	GPI BF	FPI BF	MLI BF	GPI CE	FPI CE	MLI CE	RF
Age	1	0.04	0.52	0.57	0.73	0.34	0.09	0.49	0.26
BMI	0.04	1	− 0.16	− 0.02	− 0.05	0.01	− 0.02	0.03	0.44
GPI BF	0.52	− 0.16	1	0.59	0.39	0.15	0.01	0.24	0.11
FPI BF	0.57	− 0.02	0.59	1	0.57	0.11	0.06	0.3	0.04
MLI BF	0.73	− 0.05	0.39	0.57	1	0.21	0.04	0.42	0.23
GPI CE	0.34	0.01	0.15	0.11	0.21	1	0.3	− 0.03	− 0.05
FPI CE	0.09	− 0.02	0.01	0.06	0.04	0.3	1	− 0.28	0.15
MLI CE	0.49	0.03	0.24	0.3	0.42	− 0.03	− 0.28	1	0.07
RF	0.26	0.44	0.11	0.04	0.23	− 0.05	0.15	0.07	1

*BF *biofeedback posture test, *CE* closed eyes

## Discussion

Results of this study indicate that in patients with diabetes a multicomponent exercise program, especially focused on balance exercises and gait retraining, may be an effective intervention in reducing the risk of falling and improving the functional capacity and their quality of life. To our best knowledge this is the first study to report such results.

There were studies, that proved benefit of physical therapy interventions on function of people with DPN [15]. However, there is a limited number of studies reporting on improved balance in this group [16].

In the recent study by Riandini et al. authors proved that the improvement in balance confidence was associated with a reduction in fall risk [17].

In previous studies long duration of T2D was associated with higher risk of muscle weakness and therefore more fall-related hospital stays [18, 19].

There were several studies analyzing body movement and biomechanics in patients with DPN. In comparison to healthy subjects it is characterized by a higher body swing during standing (up to 66% more), larger range of sway in the anterior–posterior and medial–lateral directions and a higher sway speed during gait [20–23]. However, more significant differences were observed when subjects were asked to perform postural stability test with eyes closed. It was explained as vision compensation of sensory deficits. Several authors underlined the significant influence of slower gait, greater stride variability and longer duration of T2D on higher risk of falls [10, 24, 25].

Besides, adults older than 70 years of age and comorbid T2D have a higher risk of sustaining more severe and potentially more demanding injuries and fractures after falls [26].

Both elderly men and women with diabetes have a higher risk of fractures than adults without diabetes, despite similar bone mineral densities [27]. This bone quality loss may be due to more severe glycosylation of amino protein groups in bone tissue, compared to other tissues, as the effect of diabetes [28]. This situation increases the risk of fractures by 64% in people with diabetes compared to healthy people [29]. It was shown that among people who were more than 65 years old and had diabetes, 30.6% fell recurrently, whereas only 19.4% of people without diabetes had recurrent falls. Recurrent falls were defined as at least 2 falls within a 6 month period [30].

The roles of aerobic exercises, resistance training and a combination of these exercises have been studied thoroughly, but the outcomes differ, even though similar exercises were performed. Morrison et al. and Read et al. concluded that these interventions improve glycemic control, overall physical function, balance and gait speed in people with diabetes [31, 32]. However, Kruse et al. stated there was no improvement in balance after a 12-month training program that included balance training exercises [33]. The explanation for this discrepancy could be the difference in the frequency and the way of executing the training program. Kruse et al. analyzed participants undergoing balance training for 11 weeks. After this initial training period, patients received regular phone calls encouraging them to continue the exercise program [33]. Allet et al. prescribed training 2 times per week for 12 weeks, for 60 min per session, in a physical therapy clinic [34].

This study showed that balance training improves postural control and clinical measures of balance. It is believed that the number and severity of diabetic complications, affects the intensive balance training outcomes.

There are few studies in which balance training programs were effectively used to improve balance and function in people with T2D. Given the likelihood of some patients having visual impairment in the course of retinopathy, further affecting their balance capabilities, these type of studies have a special relevance for the population with diabetes in establishing falling prevention programs and early treatment protocols.

## Conclusions

In patients with T2D disorders of balance and motor coordination were observed along with an increased risk of falling, compared to healthy people in similar age groups. As depicted in this study equivalent training with biofeedback improves balance and motor coordination and reduces the risk of falling in people treated for T2D. As the age of patients with diabetes increases, the static balance with vision control worsens, both before and after training sessions using the Biodex Balance System. With increasing BMI, the risk of falling of patients diagnosed with T2D was increased both before and after the biofeedback training.

## Data Availability

The datasets used and/or analyzed during the current study will be available from the corresponding author on reasonable request.

## Abbreviations

T2D: Type 2 diabetes
DPN: Diabetic peripheral neuropathy
BMI: Body Mass Index
GSI: General Stability Index
FPI: Frontal-Posterior Stability Index
MLI: Medial-Lateral Stability Index
RF: Fall risk
